# Paying Patients at Special Hospitals

**Published:** 1892-12-10

**Authors:** B. Burford Rawlings

**Affiliations:** Secretary and Director. National Hospital for the Paralysed and Epileptic, Albany Memorial, Queen Square, W.C.


### HOSPITAL OPINION.

[Contributions critical, suggestive, controversial, and technical are invited for this column, which is open to everyone who has anything to say that is of practicalvalue and interest.]

### PAYING PATIENTS AT SPECIAL HOSPITALS.

I beg your permission to assure Mr. F. Graham-Bennett, whose letter appears in your current issue, that if his statethat "the Paralytic Hospital charges from £1 1s. to £4 4s. a week to patients" is meant to apply to this institution it is singularly inaccurate.

We recognize and provide for two classes of patients only: the free, who constitute nearly five-sixths of the in-patients and all the out-patients, and "persons of the middle class of straitened means" who when admitted to in-treatment contribute one guinea weekly, or about two-thirds of their actual cost.

Now and again, but only at the express request of a member of the staff, a patient of ampler means has been admitted to a private room, borrowed for the purpose, and has paid the expense of the extra nurse or nurses. We have one such patient here now who is paying three guineas per week, and altogether we have admitted about twenty patients of this class during the past seven years.—I am, Sir, your obedient Servant,

B. Burford Rawlings, Secretary and Director.
National Hospital for the Paralysed and Epileptic,
Albany Memorial, Queen Square, W.C.,
December 5th, 1892.

